# Error in Byline

**DOI:** 10.1001/jamanetworkopen.2025.21372

**Published:** 2025-06-16

In the Invited Commentary titled “Intrinsic Capacity Centile Curves in Older Adults’ Care,”^1^ published May 12, 2025, the author listed as Regina Roller-Wirnsberger, MD, PhD, should have been listed as Regina Roller-Wirnsberger, MD, MME. This commentary has been corrected.^1^
